# Supplementary material for: The interactome of intact mitochondria by cross-linking mass spectrometry provides evidence for coexisting respiratory supercomplexes
Source: Mol Cell Proteomics. 2017 Dec 8;17(2):216–32. doi: 10.1074/mcp.RA117.000470 (PMC5795388; doi:10.1074/mcp.RA117.000470)
Supplement: Supplemental Data [file supp_RA117.000470_133922_0_supp_23978_fzffwf.zip › spectra_annotation/mito_spectra_annotation/140-1-4-1-14-1.pdf]

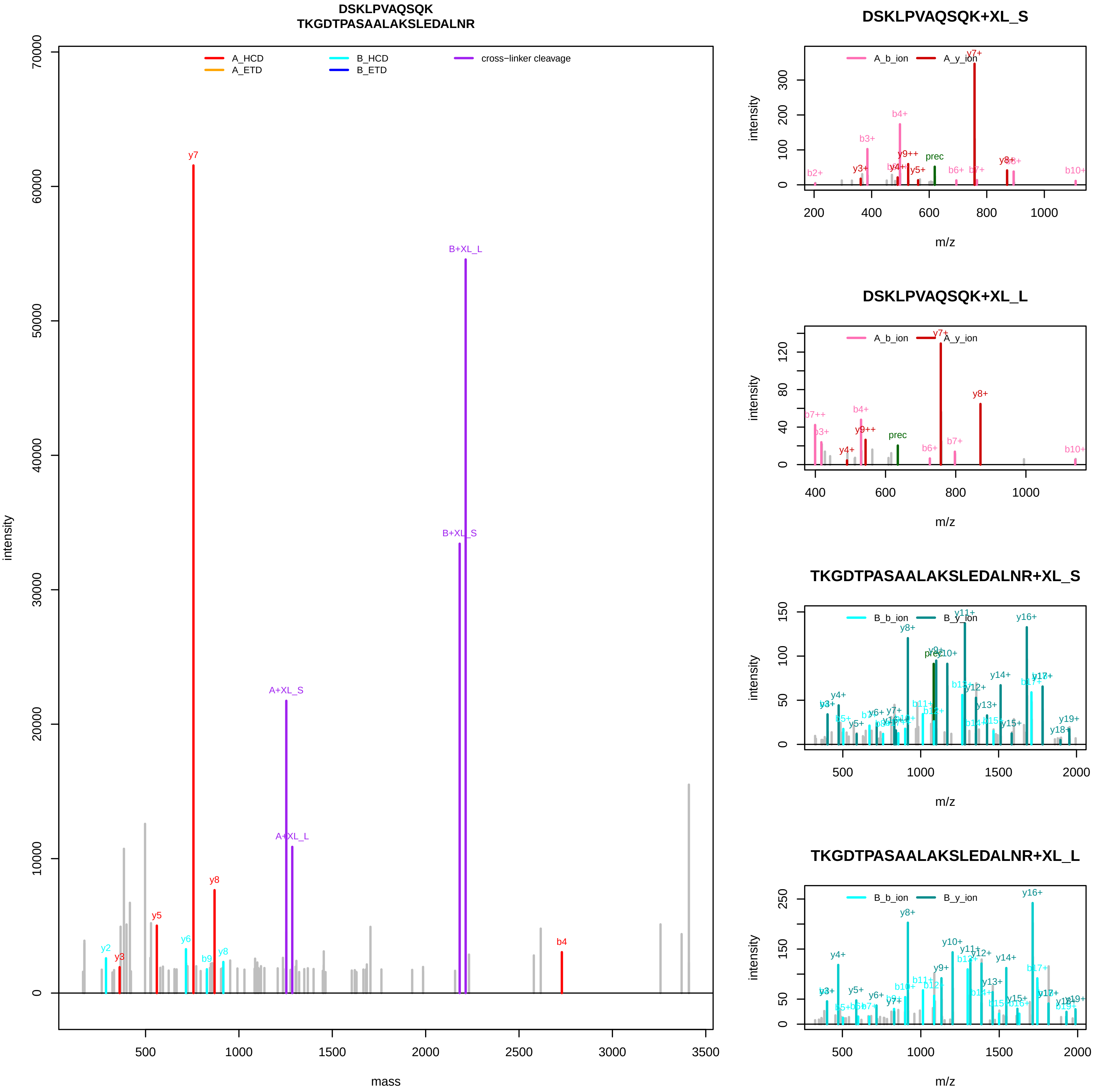

DSKLPVAQSQK
TKGDTPASAALAKSLEDALNR
A_HCD
A_ETD
B_HCD
B_ETD
cross−linker cleavage
intensity
mass
0
10000
20000
30000
40000
50000
60000
70000
500
1000
1500
2000
2500
3000
3500
y2
y3
y5
y6
b9
y7
y8
A+XL_S
A+XL_L
B+XL_S
B+XL_L
b4
DSKLPVAQSQK+XL_S
A_b_ion
A_y_ion
intensity
m/z
b2+
b3+
b4+
y3+
y9++
y5+
prec
b6+
b7+
y7+
y8+
b10+
DSKLPVAQSQK+XL_L
b7++
b3+
b4+
y4+
y9++
prec
b6+
b7+
y7+
y8+
b10+
TKGDTPASAALAKSLEDALNR+XL_S
B_b_ion
B_y_ion
y3+
y4+
y5+
y6+
y7+
y8+
prec
y11+
y12+
y13+
y14+
y15+
y16+
y17+
y18+
y19+
TKGDTPASAALAKSLEDALNR+XL_L
y3+
y4+
y5+
y6+
y7+
y8+
y9+
y10+
y11+
y12+
y13+
y14+
y15+
y16+
y17+
y18+
y19+
